# Leaf Polyphenolic Profile as a Determinant of Croatian Native Grapevine Varieties’ Susceptibility to *Plasmopara viticola*

**DOI:** 10.3389/fpls.2022.836318

**Published:** 2022-03-11

**Authors:** Petra Štambuk, Iva Šikuten, Jasminka Karoglan Kontić, Edi Maletić, Darko Preiner, Ivana Tomaz

**Affiliations:** ^1^Department of Viticulture and Enology, Faculty of Agriculture, University of Zagreb, Zagreb, Croatia; ^2^Centre of Excellence for Biodiversity and Molecular Plant Breeding, Zagreb, Croatia

**Keywords:** *Vitis vinifera* L., downy mildew, defense mechanism, leaves, chemical composition, HPLC

## Abstract

Since grapevine is highly susceptible to various pathogens, enormous amounts of pesticides are applied each season to achieve profitable production. One of the most destructive grapevine diseases is downy mildew, and their interaction has been in the spotlight for more than a decade. When it comes to a metabolome level, phenolic compounds are relevant to investigate due to their involvement in the plant immune system and known antifungal properties. Croatian grapevine germplasm is highly heterogeneous due to its long history of cultivation in diversified geographical regions. Since it has been found that native varieties react differently to the infection of *Plasmopara viticola*, the intention of this study is to define if the chemical background of the leaves, i.e., polyphenolic composition, is responsible for these dissimilarities. Therefore, the leaves of 17 genotypes, among which 14 were native and 3 were controls, were analyzed using high-performance liquid chromatography (HPLC) in four terms: before inoculation and 24, 48, and 96 h post inoculation (hpi). During this early phase, significant differences were found neither between the terms nor between the non-inoculated and inoculated samples, except for resveratrol-3-*O*-glucoside. By applying principal component analysis (PCA) using initial leaf polyphenolic composition, varieties of *V. vinifera* were clearly separated into three different groups corresponding to their International Organization of Vine and Wine (OIV) classes of susceptibility to *P. viticola*. Results obtained in this research suggest that the initial constitutive polyphenolic composition of the cultivar leaves has a crucial influence on their susceptibility to *P. viticola*, and this finding can be used to improve the success of grapevine breeding programs toward downy mildew resistance.

## Introduction

About 10,000 years of grapevine evolution and domestication in almost 90 countries (Villano and Aversano, 2020) provided a high number of genotypes possessing various morphological and genetic traits (This et al., 2006). However, the selection process carried out by humans shaped the gene pool of today’s varieties with valuable traits in the sense of yield, chemical composition, berry and bunch size, phenology, hermaphrodite flowers etc., while resistance to main pathogens was unintentionally neglected (Grassi and Arroyo-Garcia, 2020). Nowadays, it is more than ever necessary to produce enough food for the growing human population in a way that achieves a minimal footprint on the environment. That is where breeding programs play a major role and aim to bring about high-quality cultivars that can cope with the difficulties of main diseases. When it comes to the grapevine, downy mildew is one of the most destructive diseases. The causal agent of this disease is *Plasmopara viticola* [(Berk. and Curt.) Berl. and de Toni], which is one of the most damaging pathogens affecting grapevine production in all viticultural regions worldwide (Armijo et al., 2016). Diseases like mildews are allochthonous in Europe and therefore, grapevine (*Vitis vinifera* L.) varieties are susceptible to them unlike the American species [e.g., *Vitis riparia* Michx, *Vitis labrusca* L., *Vitis rupestris* Scheele, *Muscadinia rotundifolia* Small (previously *Vitis rotundifolia* Michaux)] which developed resilience coevolving on the same geographical area (Jürges et al., 2009; Gessler et al., 2011). A considerable level of resistance to downy mildew is observed in the Asian species, such as *Vitis amurensis* Rupr, which coevolved with the species of the pathogen closely related to *P. viticola*, i.e., *Plasmopara cissi* Vienn.-Bourg and *Plasmopara amurensis* Prots (Dick, 2002).

*Plasmopara viticola* is an obligate biotrophic oomycete meaning that it feeds on the living tissue, through haustoria in order to invade the host cell and obtain plant metabolites (Glazebrook, 2005). Its sporangia have lemon-shaped coenocytic cells that contain four to eight nuclei (Riemann et al., 2002). During the grapevine growing season, when conditions for downy mildew development are favorable, symptoms of infection appear on the green tissues (i.e., leaves, tendrils, inflorescences, shoots, and green berries), always starting with the young leaves. For that reason, *in vitro* experiments on the leaves are often used as an indicator of a variety’s susceptibility to *P. viticola* (Jürges et al., 2009; Bove et al., 2019). Visible adaxial symptoms on the leaves, called oil spots, are reported to usually precede the abaxial whitish sporulation (Gessler et al., 2011). When fungicides are not applied during favorable weather conditions for downy mildew development, it can devastate almost the whole yield in one season and weaken the young shoots, causing a considerable economic loss (Buonassisi et al., 2017). Yet, fungicides, both in the organic farming as copper fungicides and in the Integrated Pest Management even with other active substances, act harmfully to the environment, and animal and human health (Wilson and Tisdell, 2001); thus breeding programs aim to produce genotypes with efficient and durable resistance to main diseases, such as mildews and gray mold (Merdinoglu et al., 2018).

The mode of plant-pathogen interaction begins when the initial contact is established between infective propagules (*P. viticola* zoospores) and the plant tissue surfaces (e.g., leaf lamina). To prevent the diseases caused by pathogens, plants use sophisticated defense mechanisms that can be either constitutive or inducible defenses (Muganu and Paolocci, 2013). While the constitutive defense is referred to as a preexisting and continuous resistance (Kono and Shimizu, 2020), the induced defense is triggered by a pathogen attack and recognition and includes the perception of plant tissue signals resulting from pathogen infection (Muganu and Paolocci, 2013). Constitutive defense includes preformed physical barriers present on the plant surface (leaf hairs, wax layers, rigid cell walls, and the number and the activity of stomata) or chemical compounds, such as antimicrobial secondary metabolites (Lattanzio et al., 2006). These compounds are called phytoanticipins, which are defined as compounds that are present in plants before being challenged by microorganisms or are produced after infection solely from preexisting constituents (VanEtten et al., 1994). It has already been emphasized that increasing knowledge about constitutive phytoanticipins, such as leaf polyphenols could be pivotal to explaining the different levels of susceptibility to pathogens displayed by *V. vinifera* genotypes (Kedrina-Okutan et al., 2018). On the other hand, there are compounds that are produced by plants as a response to biotic and abiotic stresses called phytoalexins (Jeandet, 2015). Upon *P. viticola* infection of grapevine leaves, the synthesis of stilbenes is usually induced, among which resveratrol is the most common compound. It can reduce the germination of spores, which proves its strong antimicrobial activity against *P. viticola* (Dercs and Creasy, 1989). Scarce information is available suggesting that specific profiles exist at the transcriptome and metabolome level that can discriminate susceptible and resistant cultivars before being infected with *P. viticola* (Figueiredo et al., 2008).

Up to date, numerous studies have been published considering the composition and content of secondary metabolites, namely polyphenolic and volatile organic compounds, in grapevine leaves before and after *P. viticola* infection aiming to elucidate which compounds are specifically responsible for a certain level of tolerance to this microorganism among different species and varieties (Figueiredo et al., 2008, 2015; Batovska et al., 2009; Chitarrini et al., 2017; Eisenmann et al., 2019; Ciubotaru et al., 2021; Ricciardi et al., 2021). On the other hand, studies focused on the differences among *V. vinifera* varieties with different levels of resistance and their metabolomic discrimination either before or after inoculation with *P. viticola* are deficient. As a part of plants’ secondary metabolism, polyphenolic compounds and phenolic acids are not directly involved in their growth, development, and reproduction; yet they eminently participate and influence these processes. They are located in the epidermis of the leaves (cell vacuoles), cuticle, and epicuticular wax—predominantly, in the outer layers of the leaves. This epidermis/cuticle skin forms the first mechanical barrier to invading pathogens by repelling fungal spores due to its self-cleaning surface (Keller, 2020). Moreover, one of the most important roles of polyphenolics is the defense reaction due to their antifungal and antibacterial properties (Lattanzio et al., 2006). Polyphenol accumulation and profiles are influenced by seasonal climatic conditions, biotic and abiotic stressors, soil, cultural practices, and genetics (Kedrina-Okutan et al., 2018). In stressed plants, the level of reactive oxygen species (ROS) is surpassed over the antioxidant compounds. Stressors can induce the activation of the defense mechanism, which increases the biosynthesis of many phenolic compounds (Bouderias et al., 2020).

Until recently, it was thought that genetic variability among *V. vinifera* germplasm is too scarce in a sense of resistance to main fungal diseases. However, in some *V. vinifera* varieties, such as Kishmish vatkana, Dzandzal kara (Coleman et al., 2009), and Mgaloblishvili (Sargolzaei et al., 2020), resistance genes have been identified. From this kind of research, it can be concluded that varieties of local importance are possible sources of desirable features that can be useful in the upcoming changing climate as some of them are able to cope with abiotic (drought, salinity, iron chlorosis) and biotic stresses (Sargolzaei et al., 2021).

Croatia is a country with a long tradition in grapevine cultivation with many climatically diverse regions that provided to develop a high number of native grapevine varieties. The introduction of phylloxera and mildews at the end of the nineteenth century gradually caused the erosion of this preceding germplasm. Thus, today’s native collection counts slightly more than a hundred varieties (Maletić et al., 2015a; Žulj Mihaljević et al., 2020). Due to centuries-old grapevine cultivation and their adjustment to disparate environmental conditions, there is a presumption that diverse responses to diseases exist among the Croatian native varieties. These differences were recently confirmed on a series of studies applying field research, the leaf disc bioassay (OIV, 2009), and by measuring the chlorophyll fluorescence and multispectral imaging traits (Štambuk et al., 2021). In the present study, this research is extended to the metabolomic approach aiming (1) to examine the differences in the content of the polyphenols during the early stage of infection of *V. vinifera* varieties with different degree of resistance to *P. viticola* and (2) to assess the existence of a correlation between the polyphenolic profiles of 14 Croatian native *V. vinifera* genotypes, and their belonging to different classes of resistance to *P. viticola* according to the classification of the International Organization of Vine and Wine (OIV). To the best of our knowledge, up to now, there has been no research that included such a high number (15) of *V. vinifera* varieties considering their constitutive and induced leaf polyphenolic profiles regarding the level of susceptibility to *P. viticola*. Therefore, this study provides an invaluable source of information that could be used for screening other *vinifera* varieties with no defined level of susceptibility to this pathogen and to improve the success of grapevine breeding programs toward downy mildew resistance.

## Materials and Methods

### Preparation of Samples

#### Plant Material

Overall, 17 genotypes were included in this research, of which 14 were Croatian native grapevine varieties and 3 were controls. Chardonnay was used as a susceptible control variety, while Solaris and *V. riparia* are genotypes with a high and very high degree of resistance to *P. viticola*, respectively (Table 1). Chardonnay has also been used previously as a susceptible control (Deglene-Benbrahim et al., 2010; Vezzulli et al., 2018; Possamai et al., 2020). In a previous study (Štambuk et al., 2021), these genotypes were subjected to the leaf disc bioassay of *P. viticola*. According to the OIV descriptor 452-1 [Leaf: degree of resistance to *Plasmopara* (leaf disc test)], each genotype was assigned to an appropriate OIV class of resistance to downy mildew from 1, the most susceptible to 9, the totally resistant varieties (OIV, 2009). The average percentage of the *P. viticola* sporulation developed on the leaf discs of genotypes was obtained by visual scoring according to the guidelines of the European and Mediterranean Plant Protection Organization (OEPP/EPPO, 2001). Data related to OIV 053 descriptor for the young leaf, i.e., density of prostrate hairs between the main veins on the lower side of the blade are also presented in Table 1. Hardwood cuttings of the abovementioned genotypes were taken from the Croatian native grapevine varieties collection, Department of Viticulture and Enology, University of Zagreb Faculty of Agriculture in March 2019. Briefly, they were planted in regularly irrigated pots, and the shoots were grown in a greenhouse with air temperature ranging from 15 to 24°C, and relative humidity ranging from 65 to 75% during the cultivation period. In 2020, when the development of the shoots was uniform and reached a growing stage of 10 fully developed leaves (Supplementary Figure 1), the fourth and the fifth leaf beneath the apex were sampled since they do not possess age-related resistance (Steimetz et al., 2012). The leaves were transferred from the greenhouse into the laboratory and rinsed with ultrapure water. At the time of sampling in the greenhouse, the leaves were healthy with no evidence of foliar diseases.

**TABLE 1:** Genotypes, additional information on the plant material, the corresponding OIV classes of resistance of the genotypes to *P. viticola* (OIV 452-1), and the density levels of the trichomes on abaxial leaf sides (OIV 053) according to OIV (2009).

Genotype (Accession name)	Holding Institute	Material source ID (EURISCO)	VIVC code	Species	OIV 452-1	OIV 053
Belina starohrvatska	HRV041	VIT00233	5374	*Vitis vinifera* subsp. *vinifera*	1	5
Debit	HRV041	VIT00017	10423	*Vitis vinifera* subsp. *vinifera*	1	1
Grk	HRV041	VIT00030	5066	*Vitis vinifera* subsp. *vinifera*	1	3
Moslavac	HRV041	VIT00052	4292	*Vitis vinifera* subsp. *vinifera*	1	5
Plavac mali	HRV041	VIT00060	9549	*Vitis vinifera* subsp. *vinifera*	1	7
Babić	HRV041	VIT0002	844	*Vitis vinifera* subsp. *vinifera*	3	1
**Chardonnay**	HRV041	CL-277*	2455	*Vitis vinifera* subsp. *vinifera*	3	3
Kraljevina	HRV041	VIT00035	24904	*Vitis vinifera* subsp. *vinifera*	3	1
Plavina	HRV041	VIT00062	9557	*Vitis vinifera* subsp. *vinifera*	3	9
Pošip	HRV041	VIT00065	16018	*Vitis vinifera* subsp. *vinifera*	3	1
Škrlet	HRV041	VIT00085	22983	*Vitis vinifera* subsp. *vinifera*	3	3
Tribidrag	HRV041	VIT00013	9703	*Vitis vinifera* subsp. *vinifera*	3	3
Malvazija istarska	HRV041	VIT00047	7269	*Vitis vinifera* subsp. *vinifera*	5	1
Ranfol	HRV041	VIT00070	9908	*Vitis vinifera* subsp. *vinifera*	5	5
Teran	HRV041	VIT00087	12374	*Vitis vinifera* subsp. *vinifera*	5	9
**Solaris**	DEU455	20340**	20340	*Vitis vinifera* subsp. *vinifera*	7	3
***Vitis riparia***	DEU098	4609**	4609	*Vitis riparia*	9	1

*Genotypes used as controls are in bold.*

*Plant material from vineyard on Experimental station Jazbina, University of Zagreb, Faculty of Agriculture, Department of Viticulture and Enology, Cv. Chardonnay, clone CL-277.

** According to VIVC.

*VIVC—Vitis International Variety Catalog (https://www.vivc.de).*

*OIV 452-1—Descriptor for leaf: degree of resistance to Plasmopara (leaf disc test).*

*OIV 053—Descriptor for young leaf: density of prostrate hairs between the main veins on the lower side of blade.*

#### *Plasmopara viticola* Suspension Preparation

Leaves with evident *P. viticola* sporulation were taken from the naturally infected vineyard where chemical protection was not applied. In the laboratory, the leaves were soaked in ultrapure water and *P. viticola* spores were detached with a gentle brush until the water became cloudy. Prepared suspension was sprayed on the abaxial leaf sides of a susceptible variety, Chardonnay to propagate *P. viticola* spores. After 7 days, the leaves with freshly developed sporulation were soaked in ultrapure water and the sporulation was removed using a gentle brush until the suspension became dense or visibly cloudy. Suspension concentration was adjusted to 2 × 10^5^ spores ml^–1^ with Neubauer cell counting chamber (Bellin et al., 2009; Perazzolli et al., 2012; Vezzulli et al., 2018). The freshly prepared suspension was used for the inoculation of the leaves of 17 genotypes.

#### Inoculation and Incubation of the Leaves

Immediately after sampling, four leaves of each genotype were stored in the freezer at −20°C until analysis (T_0_). The remaining plant material (24 leaves per genotype) was separated into two groups, mock-inoculated leaves (treated with ultrapure water) and leaves inoculated with *P. viticola* suspension. Each leaf was placed in a separate Petri dish (150 mm in diameter) on a wet filter paper. The leaves were laid with the abaxial side up and sprayed with ultrapure water or *P. viticola* suspension. The Petri dishes were sealed with parafilm and placed in the climate chamber with optimal conditions for downy mildew development (air temperature 20°C, air moisture 80%). For the first 24 h, the samples were kept in dark, then the drops of water or suspension were removed with sterile filter paper to avoid decaying of the leaves. After that, the photoperiod of 16 h was applied to imitate the outdoor conditions (Bellin et al., 2009; Vezzulli et al., 2018). At certain time points after inoculation [T_1_—24 h post inoculation (hpi); T_2_—48 hpi; T_3_—96 hpi] (Ali et al., 2012; Chitarrini et al., 2017; Nascimento et al., 2019), the samples were taken from the climate chamber and stored in the freezer (−20°C) until analysis. During this early stage of the infectious process, the following changes in the susceptible cultivar have been previously determined: at 24 hpi, the zoospores germinate and the germ tube penetrates the substomatal cavity; at 48 hpi, the hyphae of *P. viticola* are observed in the intercellular spaces; at 96 hpi, the sporangiophores begin to develop from the stomata (Nascimento-Gavioli et al., 2020). For each genotype, the inoculation was performed on a number of leaves exceeding those necessary for the polyphenols assessment, with the aim to check the success of infection.

### Analysis of Polyphenolics

#### Extraction of Polyphenolics

Before analysis, the leaves were lyophilized (freeze-dried) and then ground using MiniG Mill (SPEX SamplePrep, United States) (1 min, 1,500 rpm) to obtain a powder. The extraction was conducted according to the method described by Sikuten et al. (2021) and Štambuk et al. (2022) with slight modifications. In brief, the solid–liquid extraction technique was performed on the magnetic stirrer (RTC basic, IKA, Staufen, Germany) in the following conditions: extraction temperature of 60°C at 400 rpm for 2 h. The mass of 40 mg of ground grapevine leaves and the volume of 3 ml of extraction solvent was used. The extraction solvent was composed of acetonitrile:water:formic acid (20:79:1, v/v/v). After extraction, each extract was filtered using a Phenex-polytetrafluorethylene (PTFE) 0.20 μm syringe filter (Phenomenex, Torrance, United States), and then analyzed by high-performance liquid chromatography (HPLC). Each sample was analyzed in triplicate.

#### High-Performance Liquid Chromatography Analysis and Identification of Compounds

The separation, identification, and quantification of polyphenolic compounds was performed on an Agilent 1100 Series system (Agilent, Waldbronn, Germany), equipped with an autosampler, a column thermostat, a diode array detector (DAD), and a fluorescence detector (FLD). The Agilent 1100 Series system is coupled to an Agilent Chem Station data-processing station. The analysis was performed according to the previously described and published method (Tomaz and Maslov, 2016). The separation was performed with a reversed-phase column Luna Phenyl-Hexyl (4.6 × 250 mm; 5 μm particle), with Phenyl guard column (4.0 × 3.0 mm) heated at 50°C. The solvents were water:phosphoric acid (99.5:0.5, v/v, eluent A), and acetonitrile:water:phosphoric acid (50:49.5:0.5, v/v/v, eluent B), and the flow rate was 0.9 ml/min. The linear gradient for eluent B was as follows: 0 min, 0%; 7 min, 20%; 35 min 40%; 40 min, 40%; 45 min 80%; 50 min, 100%; and 60, min 0%. The injection volume for each sample was 20 μl. The DAD was set to an acquisition range of 200–700 nm. Flavonol-glycosides were detected at 360 nm, hydroxycinnamic acids at 320 nm, stilbenes at 308 nm, and hydroxybenzoic acids at 280 nm using the DAD. Flavan-3-ols were detected at λ_*ex*_ = 225 nm and λ_*em*_ = 320 nm using FLD. Identification of individual flavonoids was performed by matching the retention time of each chromatographic peak with external standards and the DAD spectrum. Individual flavonoid peaks were quantified using a calibration curve of the corresponding standard compound which was based on the peak area. When reference compounds were not available, the calibration of structurally related substances was used, including a molecular weight correction factor (Kammerer et al., 2004). The results are expressed in mg/kg or g/kg of dry weight (DW) of grapevine leaves.

### Statistical Analysis

In order to define the effects of treatment (non-inoculated vs. inoculated samples), the classes of resistance and terms (time period) of sampling after inoculation, on the content of polyphenolic compounds, a factorial ANOVA was performed and the differences between the means of specific factors and their interactions were evaluated by Duncan’s multiple range test at a confidence level of 95% (*p* < 0.05) (Supplementary Table 1). However, since there was no treatment involved in the sampling term 0 (before inoculation), it was excluded from the second factorial ANOVA that was used to define the exact effects of all factors (Supplementary Table 2).

**FIGURE 1:**
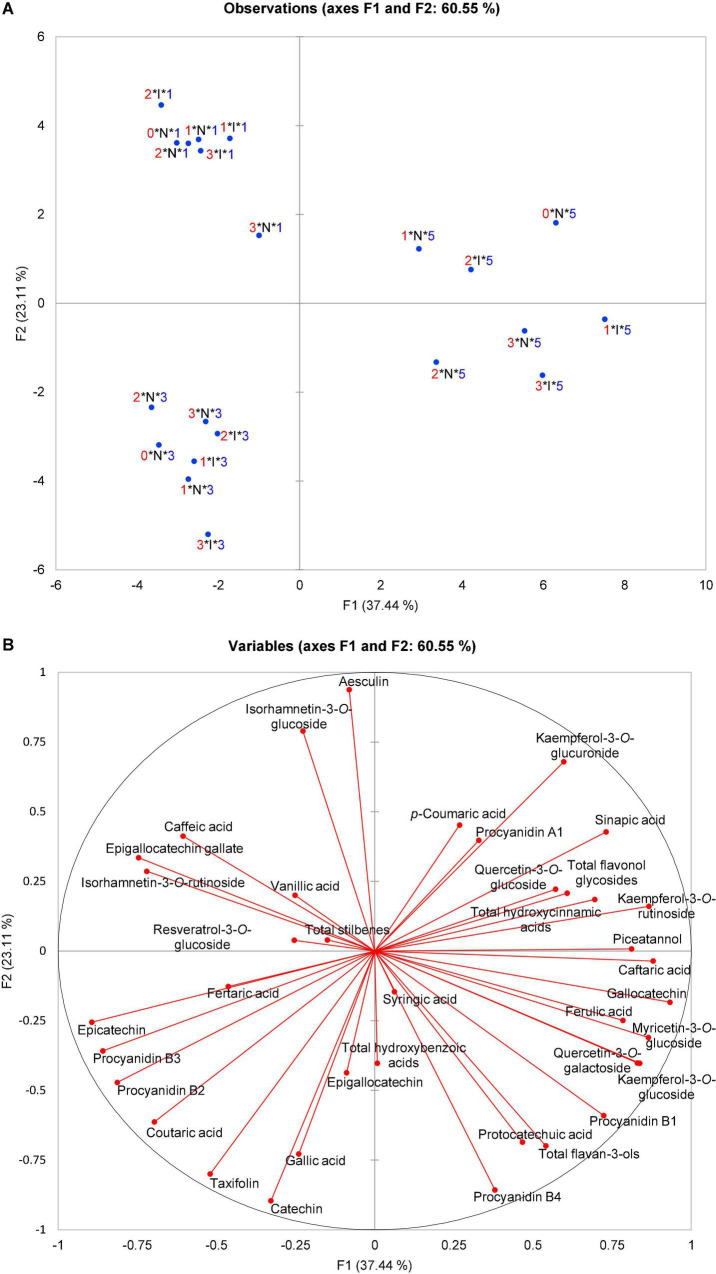
Principal component analysis (PCA) scatter plot depicting **(A)** three OIV classes of susceptibility (1, 3, and 5—*V. vinifera* varieties) based on the polyphenolic composition of their leaves before and after artificial *P. viticola* inoculation at 0, 24, 48, and 96 hpi in the space defined by the first two principal components explaining 60.55% of the variability; **(B)** the vector diagram of correlation among the content of polyphenolic compounds and the first two principal components. 0, 1, 2, 3, Terms of sampling (0, 24, 48, and 96 hpi); N, I, Non-inoculated and inoculated observations; 1, 3, 5, OIV classes of resistance.

Principal component analysis (PCA) was performed using average polyphenolic profiles of grapevine leaves for treatment (non-inoculated and inoculated), which were sampled in different terms before and upon inoculation (0, 24, 48, and 96 hpi) belonging to all genotypes used in the research (Supplementary Figure 2). Additional PCA was performed using only the average polyphenolic content of leaves within the sampling term and treatment belonging to OIV classes 1, 3, and 5 (*V. vinifera* varieties only) to focus on differences among them (Figure 1). The correlation was calculated between the data of resistance level of OIV descriptor 452-1 and the level of density of prostrate hair between the main veins on the lower side of the blade on the young leaves (OIV descriptor 053) of genotypes. Additional correlations were performed between the content of phenolic compounds and the terms of sampling separately for inoculated and non-inoculated samples (Supplementary Table 2). Correlations were calculated using Spearman’s coefficient and were tested for significance. The XLSTAT statistical and data analysis solution (Addinsoft, 2020, New York, NY, United States) was used for statistical analyses.

## Results

Among the phenolic compounds, 10 flavan-3-ols, nine flavonol glycosides, eight hydroxycinnamic acids, four hydroxybenzoic acids, and two stilbenes were detected (Supplementary Table 1). The most abundant class of phenolic compounds were hydroxycinnamic acids with an average value of 25.19 g/kg among which caftaric acid (4.99 g/kg) contributed the most. Hydroxycinnamic acids were followed by flavonol glycosides (20.2 g/kg), flavan-3-ols (5.4 g/kg), hydroxybenzoic acids (227.32 mg/kg), and stilbenes (151.72 mg/kg). As far as individual compounds are concerned, the highest amount was detected for quercetin-3-*O*-glucoside (26.39 g/kg) in the samples representing the inoculated leaves of the OIV class 3 at 48 hpi (T_2_) (Supplementary Table 1). Correlation between the content of the phenolic compounds and the terms of sampling was significant only in the case of resveratrol-3-*O*-glucoside and total stilbenes in both the inoculated and non-inoculated samples (Supplementary Table 2). There was no significant correlation found between OIV resistance classes and the density of prostrate hairs between the main veins on the lower side of the young leaves.

### Polyphenolic Profiles of Cultivars Belonging to Different International Organization of Vine and Wine Resistance Classes

A significant effect (*p* < 0.05) of OIV resistance class was observed for all 33 phenolic compounds detected in the leaves of 17 genotypes used in this research (Table 2). The effect of artificial inoculation using *P. viticola* was significant (*p* < 0.05) only in the case of compounds belonging to the group of stilbenes (piceatannol and resveratrol-3-*O*-glucoside) same as in the case of sampling term upon inoculation where one additional compound (epicatechin) was affected. There was no significant interaction of OIV classes neither with the terms of sampling nor with treatment, as well as between the terms of sampling and inoculation (Supplementary Table 2).

**TABLE 2:** The differences between OIV classes of resistance to *P. viticola* in the content of polyphenolic compounds (mg/kg dw) in the young leaves.

Polyphenolic compound (mg/kg dw)	OIV class of resistance					Polyphenolic compound (mg/kg dw)	OIV class of resistance				
			
	1	3	5	7	9		1	3	5	7	9
Myricetin 3-*O*-glucoside	284.76 bc*	341.57 b	450.71 a	246.94 bc	142.38 c	Gallic acid	0.14 b	4.56 a	1.00 b	0.00 b	0.00 b
Quercetin 3-*O*-galactoside	10.98 bc	31.32 b	67.71 a	79.83 a	0.00 c	Protocatechuic acid	120.17 ab	131.65 a	134.73 a	99.79 b	67.06 c
Quercetin 3-*O*-glucoside	21476.02 a	20459.28 a	22230.20 a	19939.34 ab	12930.30 b	Vanillic acid	37.62 c	34.33 c	31.03 c	150.65 a	85.56 b
Kaempferol 3-*O*-rutinoside	107.08 c	82.88 c	187.92 b	186.31 b	356.74 a	Syringic acid	48.08 b	47.61 b	45.10 b	6.72 c	96.87 a
Isorhamnetin 3-*O*-rutinoside	80.80 a	35.87 ab	0.00 b	0.00 b	0.00 b	*Total hydroxybenzoic acids*	206.01 a	218.15 a	211.86 a	257.16 a	249.49 a
Kaempferol 3-O-glucoside	97.56 b	119.99 ab	152.31 a	143.20 a	116.43 ab	Epigallocatechin gallate	96.61 b	69.74 bc	27.70 c	79.61 bc	510.13 a
Kaempferol 3-*O*-glucuronide	24.03 a	11.89 b	25.05 a	0.00 c	2.74 bc	Gallocatechin	602.10 b	675.72 b	1365.01 a	652.79 b	31.69 c
Izorhamnetin 3-O-glucoside	6.74 a	0.26 b	1.92 b	0.00 b	0.00 b	Epigallocatechin	1389.55 a	1607.88 a	1337.18 a	1429.44 a	313.19 b
Taxifolin	0.56 b	10.94 a	0.00 b	0.00 b	0.00 b	Procyanidin B1	2209.83 c	3019.73 b	3683.26 a	3193.26 ab	1213.24 d
*Total flavonol glycosides*	22088.52 a	21094.01 a	23115.82 a	20595.62 ab	13548.60 b	Procyanidin B3	36.81 b	48.72 a	19.65 c	40.82 ab	3.93 d
Caftaric acid	5362.67 b	5424.84 b	6101.63 a	5544.56 ab	2511.22 c	Catechin	31.36 b	60.74 a	37.21 b	54.00 a	10.87 c
Aesculin	686.79 a	352.53 b	481.52 b	227.66 b	139.39 b	Procyanidin B4	111.97 b	151.69 a	150.75 a	124.66 ab	23.30 c
Coutaric acid	120.23 b	269.23 a	69.08 b	330.28 a	217.99 ab	Procyanidin B2	133.44 b	193.71 a	65.65 c	147.83 b	49.24 c
Caffeic acid	888.08 b	840.94 b	769.88 b	1443.86 a	280.46 c	Epicatechin	391.86 b	474.72 ab	107.81 d	485.20 a	225.81 c
Fertaric acid	15.28 bc	16.19 b	14.16 bc	26.31 a	11.04 c	Procyanidin A1	82.02 a	74.12 a	82.18 a	65.61 a	26.47 b
*p*-Coumaric acid	26.71 b	20.71 c	25.66 b	12.27 d	33.83 a	*Total flavan-3-ols*	5085.54 b	6376.75 a	6876.40 a	6273.21 ab	2407.87 c
Ferulic acid	31.28 b	34.12 ab	40.94 a	41.42 a	5.44 c	Piceatannol	13.44 c	13.42 c	27.97 b	37.79 b	63.97 a
Sinapic acid	3633.00 a	3213.18 b	3801.47 a	3372.13 ab	2154.91 c	Resveratrol 3-*O*-glucoside	183.21 a	143.12 b	101.44 c	70.09 c	208.62 a
*Total hydroxycinnamic acids*	27451.19 a	26518.85 a	29217.45 a	26140.19 a	16059.81 b	*Total stilbenes*	196.64 b	156.54 c	129.41 cd	107.88 d	272.59 a

*Means were evaluated by Duncan’s multiple range test at a confidence level of 95% (p < 0.05). Different letters show statistical significance.

Comparing the mean values of individual phenolic compounds within the different OIV classes of resistance, especially classes 1, 3, and 5 involving *V. vinifera* cultivars (Table 2), the most abundant compounds detected in class 5 were the following: myricetin-3-*O*-glucoside, quercetin-3-*O*-galactoside, quercetin-3-*O*-glucoside, kaempferol-3-*O*-glucoside, caftaric acid, gallocatechin, procyanidin B1, and piceatannol. The content of myricetin-3-*O*-glucoside and gallocatechin were significantly the highest in OIV 5, whereas the contents of the remaining mentioned compounds were not statistically different from OIV 7. Three varieties belonging to OIV 5 showed variations in the content of these compounds, especially the variety, Teran which showed the highest concentration of procyanidin A1 and caftaric acid (Supplementary Table 3).

Class 1 represents the most susceptible group of varieties (Table 1). By comparing the OIV class 1 with classes 3 and 5, significantly higher contents were detected for isorhamnetin-3-*O*-glucoside, aesculin, resveratrol-3-*O*-glucoside, and the total content of stilbenes, whereas the least detected were procyanidin B1, procyanidin B4, and the content of total flavan-3-ols (Table 2).

Varieties belonging to the OIV class 3, including the control variety, Chardonnay, had a significantly higher content of taxifolin, coutaric acid, gallic acid, catechin, procyanidins B2 and B3 when compared to OIV classes 1 and 5. No significant differences between these three classes were detected for quercetin-3-*O*-glucoside, caffeic acid, fertaric acid, protocatechuic acid, vanillic acid, syringic acid, epigallocatechin, and procyanidin A1, total flavonol glycosides, hydroxycinnamic, and hydroxybenzoic acids (Table 2). Varieties belonging to OIV 3 have similar profiles of polyphenolic compounds except for the varieties, Pošip and Plavina. Pošip has a high content of caftaric acid, and resveratrol 3-*O*-glucoside, whereas in both of them, high content of procyanidin A1 is detected (Supplementary Table 3 and Supplementary Figure 2).

**FIGURE 2:**
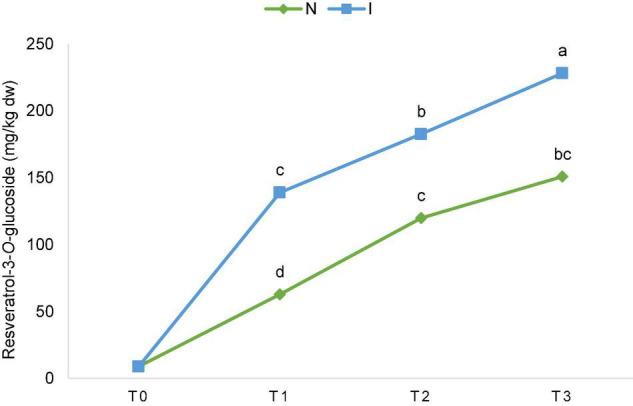
The ascending content of resveratrol-3-*O*-glucoside throughout the experiment [before inoculation (T_0_), 24 hpi (T_1_), 48 hpi (T_2_), and 96 hpi (T_3_)] for non-inoculated (N) and inoculated (I) samples regardless of the International Organization of Vine and Wine (OIV) class. The values for each time point and treatment were obtained by the mean of the values of 17 genotypes. The differences between the means were evaluated by Duncan’s multiple range test at a confidence level of 95% (*p* < 0.05). Different letters show statistical significance.

Significant effect of inoculation on the content of piceatannol and resveratrol-3-*O*-glucoside was detected. Differences between the inoculated and non-inoculated samples were already significant in T_1_ (24 h after inoculation) and continued through T_2_ and T_3_ for resveratrol-3-*O*-glucoside (Figure 2), while for piceatannol, besides the overall significant effect in factorial ANOVA, differences within the terms were not significant. Consequently, the total content of stilbenes was also significantly different since this group of polyphenolics is comprised of piceatannol and resveratrol-3-*O*-glucoside only (Supplementary Table 2). The ascending content of resveratrol-3-*O*-glucoside throughout the experiment is depicted in Figure 2 for non-inoculated (N) and inoculated (I) samples regardless of the OIV class. Upon inoculation, the ascending content between the terms of sampling was significant for flavan-3-ol epicatechin (Supplementary Table 2).

### Discrimination of *Vitis vinifera* Varieties

The focus of this study was on the variability of leaf polyphenolic compounds related to the difference of *V. vinifera* varieties. Therefore, a PCA was performed to analyze the total variability of the polyphenolic composition of the leaves before and after artificial *P. viticola* inoculation at 0, 24, 48, and 96 hpi that belong to OIV classes 1, 3, or 5 (*V. vinifera* varieties). Mock-inoculated (control, non-inoculated) leaves were sampled throughout the experiment simultaneously with inoculated ones. The PCA scatter plot of the first two components explained 60.55% of the variability (Figure 1) with the first principal component (PC1) accounting for 37.44% and the second (PC2) for 23.11%. Projection on these two axes separated the samples into three groups corresponding to three OIV classes (1, 3, and 5), whereas the infection status of the samples (non-inoculated (N) or inoculated (I)), and the terms of sampling were not separated (Figure 1A).

Based on the vector diagram (Figure 1B), it is possible to define the phenolic compounds that contributed to such distribution and grouping of samples belonging to different OIV classes in the space defined by the first two principal components. A group containing all the samples belonging to OIV class 5 regardless of the treatment and the sampling term was separated from the other two groups mainly based on the higher content of quercetin-3-*O*-glucoside, myricetin-3-*O*-glucoside, kaempferol-3-*O*-rutinoside, piceatannol, caftaric acid, ferulic acid, and gallocatechin together with the contents of total flavonol glycosides and hydroxycinnamic acids. As for the group containing all the samples belonging to OIV class 3, all the observations are in the third quadrant and almost diametrically opposed to OIV class 5. The position of this group was defined mainly by a higher content of catechin, epicatechin, epigallocatechin, taxifolin, coutaric and gallic acid, and procyanidins, B2 and B3. Class 1 is distinguished by a higher content of isorhamnetin-3-*O*-glucoside, isorhamnetin-3-*O*-rutinoside, caffeic and vanillic acid, epigallocatechin-gallate, resveratrol-3-*O*-glucoside, and by the content of total stilbenes.

## Discussion

Studies considering metabolomic changes of the grapevines and profiling regarding the different levels of susceptibility to *P. viticola* have been intriguing for more than a decade and this trend does not seem to fade. For this purpose, HPLC proved to be a reliable and scrutinized analytical technique by which it is possible to quantify phenolic acids and polyphenolic compounds (Tomaz and Maslov, 2016). As possible progress of breeding programs that are oriented toward improved resistance, *V. vinifera* varieties are in the spotlight to research and use as progenitors since most of them do not contain undesirable viticultural and oenological features like American species (Toepfer et al., 2011).

Plant metabolites can be either included in the primary metabolism, such as lipid compounds, amino acids, and sugars, or secondary metabolism, such as phenolic compounds arising biogenetically from the shikimate-phenylpropanoids-flavonoids pathways (Lattanzio et al., 2006). Some of the important physiological roles of phenolic compounds are the formation of the cell wall polymers, lignin (Paone et al., 2020) and suberin (Bernards and Razem, 2001), floral and fruit pigment synthesis (Tanaka et al., 2008), ultraviolet sunscreen protection (Cefali et al., 2016), formation of flavor compounds (Kielhorn and Thorngate, 1999), and defense against pathogens (Zaynab et al., 2018).

Gómez-Zeledón and Kaiser (2016); Buonassisi et al. (2018), and Vezzulli et al. (2018) have proposed OIV descriptor 452-1 (OIV, 2009), and therefore it was also applied in the research aiming to distinguish groups of resistance among Croatian native grapevine varieties as this method is reliable with included control genotypes (Štambuk et al., 2021). Nonetheless, for comprehensive research purposes, in the present work, the screening is extended and dedicated to the analysis of secondary metabolites, namely polyphenolic compounds and phenolic acids aiming to define their possible relation with differences in the resistance level among them.

The most abundant group of polyphenolic compounds detected in the leaves of genotypes included in this study were hydroxycinnamic acids (25.19 g/kg DW) followed by flavonol glycosides, whose content was slightly lower (20.2 g/kg DW). Flavonol glycosides have photoprotective roles by filtering the UV-B light while allowing to pass photosynthetically active visible light (Agati et al., 2013) together with an antioxidant function during plant response to environmental stress (Hernández et al., 2009). Quercetin-3-*O*-glucoside was the most abundant flavonol in the research performed by  Anđelković et al. (2015) in the grapevine leaf extracts from Vranac and Merlot (*V. vinifera*). Previous studies confirmed the higher susceptibility of shaded leaves grown in the greenhouse that contains less flavonols (Agati et al., 2008; Latouche et al., 2013) thus supporting the results of the present study where hydroxycinnamic acids are the most abundant polyphenolic group, probably due to reduced ultraviolet radiation conditions in the greenhouse. According to Meyer et al. (2021), supplemental UV-B light has a positive effect on disease resistance in many plant-pathogen combinations, mainly through the induction of the production of specialized metabolites like flavonoids.

No significant differences were found between the control and the inoculated leaves regardless of the OIV class and the term of sampling, with the exception of stilbenes and resveratrol-3-*O*-glucoside specifically. That is in accordance with the previous study where non-destructive optical methods were used (Latouche et al., 2013) for monitoring flavonols, hydroxycinnamic acids, and stilbenes throughout 6 days upon *P. viticola* inoculation in the Cabernet Sauvignon leaves grown in the greenhouse. Resveratrol-3-*O*-glucoside also showed a significant positive correlation in terms of sampling both in the case of inoculated and non-inoculated samples. Stilbenes are the most important class of phytoalexins in the *Vitaceae* family, which are dynamically accumulated in response to various abiotic and biotic stresses, including pathogen attacks (Ciaffi et al., 2019). Thus, their ascending content in the non-inoculated leaves could be explained by picking them as well as changing the environment from the greenhouse to the laboratory conditions. Moreover, inoculation with *P. viticola* suspension caused additional stress and thus in these leaves, the contents of resveratrol-3-*O*-glucoside and total stilbenes were even higher throughout the experiment. Previous studies have shown that the accumulation of higher stilbenes is usually associated with the response of the resistant genotypes to *P. viticola* infection (Boso et al., 2012; Chitarrini et al., 2017). In our study, huge variability was detected among the genotypes belonging to different resistance classes confirming that the accumulation of stilbene resveratrol-3-*O*-glucoside is related to infection. However, the obtained results also suggest that increased content of resveratrol-*3-O*-glucoside is not sufficient to achieve a high level of resistance to *P. viticola.* In response to the presence of *P. viticola*, stilbenes are synthesized in grapevine leaves (Chalal et al., 2014). They possess antimicrobial activity that may be strong enough to inhibit the infection in resistant genotypes (Chong et al., 2009), which is not accurate for susceptible genotypes. Based on the previous studies, the content of stilbenes increases in accordance with *P. viticola* development in susceptible varieties suggesting that the accumulation of stilbenes can be used as an indicator of *P. viticola* infection (Naidenov et al., 2010; Latouche et al., 2013).

In the present work, three susceptible OIV classes (1, 3, and 5) are distinguished by each group characteristic of polyphenolic compounds provided by PCA. More specifically, the most susceptible OIV class 1 was separated from two other groups by being abundant in caffeic and vanillic acid, which are hydroxycinnamic and hydroxybenzoic acids, respectively. Caffeic acid has also been found in high amounts in susceptible *V. vinifera* varieties (Riesling Weiss, Pinot Noir, Cabernet Sauvignon, and Trincadeira) (Maia et al., 2020). On the contrary, caffeic acid has been previously related to constitutive resistance in the partially resistant cultivar Regent (Figueiredo et al., 2008). This compound participates in enzymatic oxidative mechanisms in response to the pathogenic infection of the grapevine (Mattivi et al., 2011). Among flavan-3-ols, the only discriminator was epigallocatechin-gallate known for its high antioxidant capacity (Kedrina-Okutan et al., 2018).

Flavan-3-ols, i.e., catechin and epicatechin, were more abundant in the presented OIV class 3. A previous study (Maia et al., 2020) hypothesizes that higher levels of catechin/epicatechin and over-expression of *LAR2* gene (involved in the conversion of leucocyanidin into catechin and epicatechin) may be putative biomarkers of susceptibility. Catechin, together with other phenolic compounds, possesses antioxidant properties and has been previously determined as a part of the grapevine defense mechanism (Kortekamp, 2006). However, there is a presumption that catechin can be degraded by different fungi, used as a carbon source for growth, and finally used for establishing a successful infection (Maia et al., 2020), but the precise potential of *P. viticola* in the degradation of this compound is not investigated. Epicatechin has been proposed as a biomarker of resistance in a study by Ciubotaru et al. (2021) due to its higher content in the genotype, BC4 possessing resistant locus *Rpv1*. Phenolic acids, namely fertaric, coutaric, and gallic acid, have also contributed to the discrimination. Nevertheless, Ali et al. (2012) identified fertaric acid in the partially resistant cultivar Regent.

Quercetin-3-*O*-glucoside was a discriminative compound that was more abundant in the OIV class 5, which is in accordance with a previous study (Maia et al., 2020) where the same flavonol glycoside together with several others was found in higher concentrations in the resistant/partially resistant genotypes. Another flavonol, namely kaempferol-3-*O*-rutinoside that distinguished this class, was also detected previously in the partially resistant cultivar, Bianca at 12 hpi (Chitarrini et al., 2017). Furthermore, quercetin-3-*O*-glucoside and caftaric acid were found at higher concentrations and therefore were responsible for distinguishing Regent from Trincadeira (Ali et al., 2012). Latouche et al. (2013) observed that constitutive higher content of flavonols slowed down the accumulation of stilbenes in the grapevine leaves, and thus the phytoalexin-mediated response of leaves to *P. viticola* was delayed, suggesting that constitutive higher amounts of flavonols could confine the spreading of the pathogen. *Trans*-caftaric acid was the most abundant phenolic acid in the leaf extracts of Vranac and Merlot, with lower content in infected ones ( Anđelković et al., 2015). Ferulic and *p*-coumaric acids were also discriminative for the OIV 5. The highest contents of these acids were previously found in the interspecies hybrid Petra, with 12.5% of *Vitis amurensis* and 87.5% of *Vitis vinifera* in its genetic background, among other pure *V. vinifera* varieties (Pantelić et al., 2017). Petra is known for high cold hardiness and reduced susceptibility to *P. viticola* and *Botrytis cinerea* (Cindric et al., 2003).

Apart from constitutive and induced chemical compounds that provide a certain level of tolerance to parasitic microorganisms, resistance to *P. viticola* can be associated with the synthesis of physical barriers, such as callose and lignin appositions (Toffolatti et al., 2012). Moreover, hydrophobic trichomes on the abaxial leaf sides reduce the retention or repel water drops, thus preventing the encystment of *P. viticola* zoospores (Kono and Shimizu, 2020), a step that is essential for the pathogen development inside a leaf tissue and further fructification (Rossi and Caffi, 2007, 2012). This morphological feature is an example of passive resistance, whereas active responses involve hypersensitivity and synthesis of specific secondary metabolites (Buonassisi et al., 2017). The morphological characteristic of Croatian native varieties, i.e., Teran and Ranfol, is abaxial leaf sides covered by extremely dense hydrophobic and moderately dense trichomes, respectively, that certainly obstruct *P. viticola* sporangia to reach the epidermis and stoma at the leaf bottom. On the other hand, the leaves of Malvazija istarska are glabrous; yet they are firm and robust (Maul et al., 2012; Maletić et al., 2015a) whose possibly thick cuticle protect them from plant pathogens (Serrano et al., 2014). There are varieties with a relatively high density level of trichomes within the classes of resistance, such as Belina starohrvatska, Moslavac, and Plavac mali in class 1, Plavina in class 3, and Ranfol and Teran in class 5. Opposed to this, in the case of resistant genotypes (class 7 and 9), low density levels of the trichomes are present. Subsequently, no correlation between the density of the trichomes and resistance to *P. viticola* was determined suggesting that this feature does not have a major effect on the resistance level of specific genotypes, in contrast to some previous studies (Kortekamp and Zyprian, 1999; Kono and Shimizu, 2020).

Solaris, one of the control varieties used in this research, proved its high yet not complete resistance to *P. viticola* (OIV 452 ≈ 7) in previous studies (Vezzulli et al., 2018; Ciubotaru et al., 2021) since this variety contains two resistance genes (*Rpv3-3* and *Rpv10*) (Vezzulli et al., 2019; Possamai et al., 2020). Such a pyramided resistance provides a higher level of resistance generally expressed as a more stable and durable feature (Merdinoglu et al., 2018). However, it was found that its response to *P. viticola* infection is isolate-specific and highly variable (Heyman et al., 2021). Due to its genetic background based on *V. vinifera* [Merzling × (Zarya Severa × Muscat Ottonel)] (Pezet et al., 2004), it reacted more similarly to *V. vinifera* varieties, when compared to *V. riparia* upon *P. viticola* inoculation. This is comparable with previous research where Regent’s (on which backcrosses were made with *V. vinifera*) metabolic profile clustered together with *V. vinifera* varieties (Maia et al., 2020). In Bianca, which has an *Rpv3* locus in its genome, the content of the secondary metabolites increased at later stages after the infection (96 hpi). These were phenylpropanoids, flavonols, and stilbenes, whereas the earliest modifications included primary metabolites, i.e., lipids, amino acids, acids, and sugars at 24–48 hpi (Chitarrini et al., 2017).

*Vitis riparia* is an indigenous species to North America where it evolved with fungi/oomycete, *E. necator* and *P. viticola*, and subsequently developed resistance to mildew diseases (OIV 452 = 9). Low or no sporulation values were associated with this genotype in the previous studies (Boso et al., 2012; Bhattarai et al., 2021). Thus, it has been effectively used in breeding programs for resistance introgression (Toepfer et al., 2011). Upon *P. viticola* infection, this genotype produced the highest content of resveratrol-3-*O*-glucoside, piceatannol, and total stilbenes which have been observed previously (Boso et al., 2012) due to the fast constitutive expression of the stilbene synthase genes as well as the extent of their transcriptional activation following *P. viticola* inoculation (Ciaffi et al., 2019). Stilbenes are toxic to phytopathogenic fungi and may contribute to disease resistance as phytoalexins (Ribera and Zuñiga, 2012). Although stilbenes were also identified in susceptible genotypes, they contributed to the differentiation of the OIV class 1; their importance is much greater in discriminating the resistant genotype. Along with stilbenes, epigallocatechin-gallate and kaempferol-3-*O*-rutinoside discriminated the OIV class 9 from all other OIV classes. Kedrina-Okutan et al. (2018) stated that *V. riparia* leaves constitutively contain a higher amount of total polyphenols, total flavonols, and total phenolic acids compared to *V. rupestris*, which could explain its specifically high resistance to *P. viticola*, as the flavonols limit this pathogen development (Ali et al., 2012). Comparing metabolic compositions associated with disease susceptibility of different *Vitis* species and *V. vinifera* varieties, *V. riparia* clustered together with *V. labrusca*, *V. candicans*, *V. vinifera* subsp. *sylvestris*, and *V. rotundifolia*, whereas the Regent was closer to *V. vinifera* varieties, such as Riesling Weiss and Pinot Noir (Maia et al., 2020), confirming the results of the present study. The susceptible control variety, Chardonnay was previously included in a study (Toffolatti et al., 2012) where the changes of antifungal compounds upon *P. viticola* infection are described and flavonoids showed no specific reaction to the presence of this pathogen. In our study, the polyphenolic profile of Chardonnay was similar to most of the other genotypes belonging to OIV class 3, although some compounds (i.e., protocatechuic acid, gallocatechin, procyanidins B1, B3, and B4) were higher than in the case of other genotypes from this class. Chardonnay and the native variety, Kraljevina were specific for the highest content of gallic acid.

There were no previous studies on the polyphenolic composition of the leaves of Croatian native varieties used in this study and our previous study (Štambuk et al., 2021) was the first one on the susceptibility of Croatian grapevine germplasm. Results from both of these studies, confirm the high level of variability within the Croatian native varieties previously defined for other important characteristics (Maletić et al., 2015b).

## Conclusion

This work has demonstrated the importance of secondary metabolites in grapevine defense responses against *P. viticola* with particular emphasis on Croatian native varieties. The research is based on a detailed analysis of phenolic compounds responsible for the discrimination of varieties among the OIV classes of resistance. The performed polyphenolic analysis confirmed and fulfilled the previous studies suggesting that constitutive polyphenolic profile contributes to the separation of susceptible OIV classes (1, 3, and 5) into three groups. The high variability in the content of resveratrol-3-*O*-glucoside and total stilbenes was determined, and discrimination among non-infected and infected samples was detected. However, the content of this compound did not show a clear difference between the resistant and susceptible genotypes. The content of piceatannol and total stilbenes discriminated completely resistant OIV class 9 (*V. riparia*) and the remaining OIV classes, thus confirming their strong antimicrobial properties. Considering the polyphenolic profiles of *V. vinifera* varieties, mostly flavonol glycosides were found to be responsible for lower susceptibility. Multivariate analysis shows complex relations among phenolic profiles and resistance levels suggesting that the preinfectional phenolic profile of leaves could be a determinant for different susceptibility to *P. viticola.*

Less susceptible grapevine varieties that belong to OIV class 5 (Malvazija istarska, Ranfol, Teran) could be interesting to use in breeding programs aiming to produce high-quality genotypes resistant to main fungal diseases. A further intention is directed toward analyzing constitutive and induced volatile organic compounds since their profile should distinguish grapevine classes of susceptibility to *P. viticola* likewise, whereas potential early metabolomic changes should elucidate additional bioactive molecules.

## Data Availability Statement

The original contributions presented in the study are included in the article/Supplementary Material, further inquiries can be directed to the corresponding author/s.

## Author Contributions

PŠ, JK, and IT contributed to the conceptualization of the study. PŠ and IT contributed to the methodology. PŠ, IŠ, and DP conducted the formal analysis. PŠ and IŠ performed the investigations. DP performed data curation. PŠ wrote the original draft preparation. IŠ, DP, JK, EM, and IT contributed to the writing, reviewing, and editing. JK and IT supervised the work. EM contributed to funding acquisition. All authors contributed to the manuscript revision, read, and approved the submitted version.

## Conflict of Interest

The authors declare that the research was conducted in the absence of any commercial or financial relationships that could be construed as a potential conflict of interest.

## Publisher’s Note

All claims expressed in this article are solely those of the authors and do not necessarily represent those of their affiliated organizations, or those of the publisher, the editors and the reviewers. Any product that may be evaluated in this article, or claim that may be made by its manufacturer, is not guaranteed or endorsed by the publisher.

## Supplementary Material

The Supplementary Material for this article can be founrd online at: https://www.frontiersin.org/articles/10.3389/fpls.2022.836318/full#supplementary-material

Supplementary Figure 1 Genotypes in the greenhouse.

Supplementary Figure 2 PCA_Single genotypes and polyphenolic compounds.
