# Supplementary figures and images for: Leaf Polyphenolic Profile as a Determinant of Croatian Native Grapevine Varieties’ Susceptibility to Plasmopara viticola
Source: Front Plant Sci. 2022 Mar 11;13:836318. doi: 10.3389/fpls.2022.836318 (PMC8963502; doi:10.3389/fpls.2022.836318)

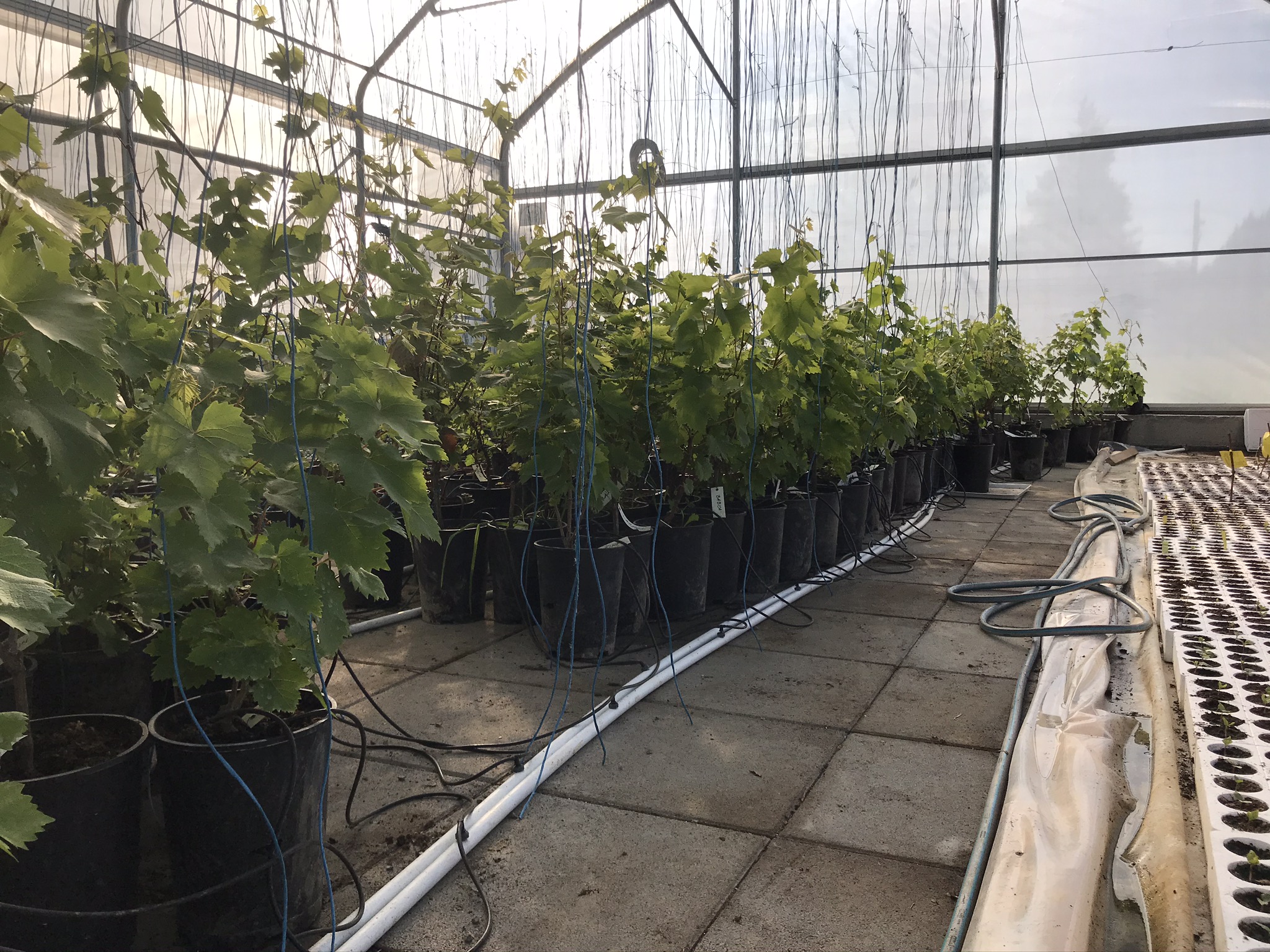

Supplement: Supplementary Figure 1 — Genotypes in the greenhouse. [file Image_1.JPEG]

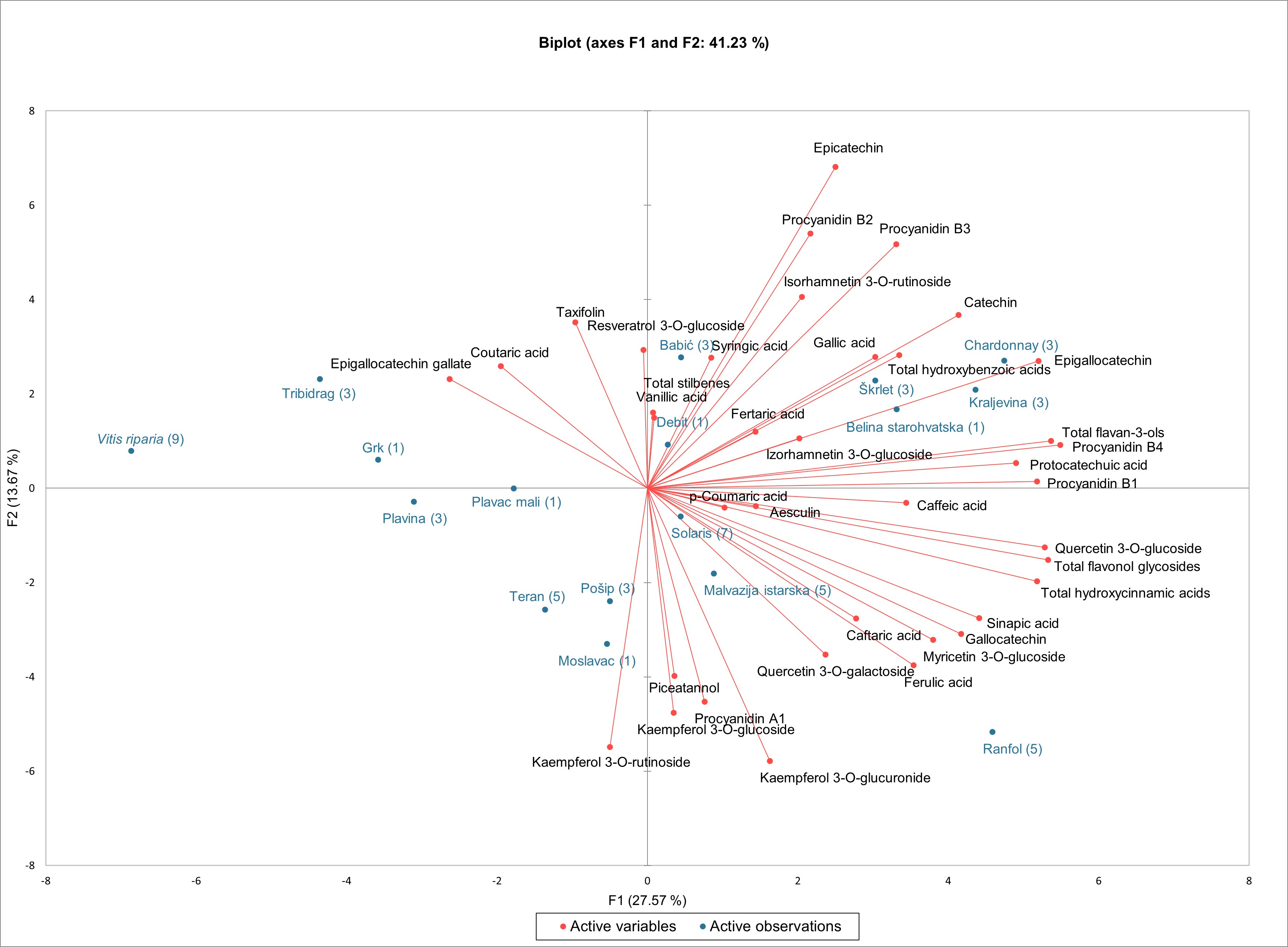

Supplement: Supplementary Figure 2 — PCA_Single genotypes and polyphenolic compounds. [file Image_2.JPEG]
